# Development of an *in vitro* compound screening system that replicate the *in vivo* spine phenotype of idiopathic ASD model mice

**DOI:** 10.3389/fphar.2024.1455812

**Published:** 2024-08-29

**Authors:** Kazuma Maeda, Miki Tanimura, Yusaku Masago, Tsukasa Horiyama, Hiroshi Takemoto, Takuya Sasaki, Ryuta Koyama, Yuji Ikegaya, Koichi Ogawa

**Affiliations:** ^1^ Laboratory for Drug Discovery and Disease Research, Shionogi Pharmaceutical Research Center, Shionogi and Co., Ltd., Osaka, Japan; ^2^ Laboratory of Chemical Pharmacology, Graduate School of Pharmaceutical Sciences, The University of Tokyo, Tokyo, Japan; ^3^ Department of Pharmacology, Graduate School of Pharmaceutical Sciences, Tohoku University, Sendai, Japan

**Keywords:** autism spectrum disorder, dendritic spine, phenotypic screening, 5-HT receptor modulator, BTBR T+ Itpr3tf/J

## Abstract

Autism Spectrum Disorder (ASD) is a developmental condition characterized by core symptoms including social difficulties, repetitive behaviors, and sensory abnormalities. Aberrant morphology of dendritic spines within the cortex has been documented in genetic disorders associated with ASD and ASD-like traits. We hypothesized that compounds that ameliorate abnormalities in spine dynamics might have the potential to ameliorate core symptoms of ASD. Because the morphology of the spine is influenced by signal inputs from other neurons and various molecular interactions, conventional single-molecule targeted drug discovery methods may not suffice in identifying compounds capable of ameliorating spine morphology abnormalities. In this study, we focused on spine phenotypes in the cortex using BTBR *T* ^*+*^ *Itpr3* ^*tf*^/J (BTBR) mice, which have been used as a model for idiopathic ASD in various studies. We established an *in vitro* compound screening system using primary cultured neurons from BTBR mice to faithfully represent the spine phenotype. The compound library mainly comprised substances with known target molecules and established safety profiles, including those approved or validated through human safety studies. Following screening of this specialized library containing 181 compounds, we identified 15 confirmed hit compounds. The molecular targets of these hit compounds were largely focused on the 5-hydroxytryptamine receptor (5-HTR). Furthermore, both 5-HT_1A_R agonist and 5-HT_3_R antagonist were common functional profiles in hit compounds. Vortioxetine, possessing dual attributes as a 5-HT_1A_R agonist and 5-HT_3_R antagonist, was administered to BTBR mice once daily for a period of 7 days. This intervention not only ameliorated their spine phenotype but also alleviated their social behavior abnormality. These results of vortioxetine supports the usefulness of a spine phenotype-based assay system as a potent drug discovery platform targeting ASD core symptoms.

## Introduction

Autism Spectrum Disorder (ASD) represents a developmental disorder characterized primarily by challenges in social interaction, communication impairments, and restricted, repetitive behaviors (Lord et al., 2018). Currently, only two medications—risperidone and aripiprazole—are approved by the US Food and Drug Administration (FDA) for ASD, specifically for alleviating irritability symptoms (Lamy and Erickson, 2018). These drugs offer limited relief and there exists no FDA-approved treatment targeting the core symptoms of ASD. Selective Serotonin Reuptake Inhibitors (SSRIs) are sometimes prescribed off-label to address anxiety, mood disturbances, and irritability in ASD, yet their effectiveness remains inconclusive (Williams et al., 2010; Aishworiya et al., 2022).

Efforts to identify effective agents for ASD core symptoms through drug repurposing have yielded limited success (Aishworiya et al., 2022; Koch and Demontis, 2022; Fernell et al., 2021; Yamasue et al., 2018; Dy et al., 2017). To overcome this challenge, it is crucial to gain a comprehensive understanding of ASD pathology and mechanisms, utilizing this knowledge to drive targeted drug discovery.

ASD etiology encompasses genetic and environmental factors, and their interplay should also be considered (Chaste and Leboyer, 2012). Genetic factors contribute significantly, being involved in 60% to more than 90% of cases, and the involvement of more than 100 genes has been reported (Bai et al., 2019; Betancur, 2011). A collection of genetic disorders such as Fragile X syndrome (FXS), Rett syndrome, and Angelman syndrome present shared ASD-like symptoms and dendritic spine abnormalities within excitatory synapses in the cortex (Hutsler and Zhang, 2010; Irwin et al., 2001; Phillips and Pozzo-Miller, 2015; Martínez-Cerdeño, 2016). These genetic disorders may manifest ASD-like symptoms due to compromised synaptic function associated with abnormal dendritic spines in the cortex—a region pivotal for cognitive functions, including social interactions (Irwin et al., 2001).

While not all ASD symptoms can be attributed to dendritic spine abnormalities, several monogenic animal models lacking specific genes have displayed associations between ASD-like behavior and abnormal spine morphology. For instance, FXS, caused by mutations in the *FMR1* gene, is marked by heightened spine density and an increased proportion of immature spine forms in patients (Irwin et al., 2001). Correspondingly, *FMR1* knockout (KO) mice—a model of FXS—exhibit elevated spine density and a higher density of elongated, immature spines in specific cortical regions (Comery et al., 1997; Hodges et al., 2017). Additionally, ASD-like behavior and concurrent spine morphology anomalies have been observed in various transgenic mouse models featuring ASD-related genes found in patients (Hulbert and Jiang, 2016).

However, relying solely on monogenic models to identify drug candidates targeting ASD phenotypes risks the development of treatments effective solely in individuals with specific genetic backgrounds. To circumvent this, we employed BTBR *T* ^*+*^ *Itpr3* ^*tf*^/J (BTBR) mice, which are used as an idiopathic model in non-clinical ASD research and exhibit consistent and robust autism-like behaviors (Moy et al., 2007; Scattoni et al., 2008; Meyza and Blanchard, 2017). Transcriptome analyses of their cortex and hippocampus revealed altered gene expression patterns associated with synaptic transmission and actin cytoskeleton regulation (Daimon et al., 2015; Kratsman et al., 2016). Based on these findings, we postulated that the regulation of synaptic skeletal structures, namely, dendritic spines, might be impaired in BTBR mice. Indeed, previous reports have indicated a reduced proportion of mature spines and an increased presence of immature spines in the cerebellum of BTBR mice (Xiao et al., 2020).

In this study, we focused on dendritic spine morphology within the cortex and identified abnormalities in BTBR mice. Based on these findings, we hypothesized that compounds capable of modulating spine morphological aberrations could potentially alleviate ASD-like behaviors in this model. We replicated the observed spine phenotype in cultured neurons *in vitro* and performed phenotypic compounds screening. Subsequently, identified compounds were evaluated *in vivo*, revealing their potential to ameliorate spine abnormalities and social behavioral impairments, but not repetitive behavior, in BTBR mice.

## Materials and methods

### Animals

Animal experiments adhered to the NIH guidelines for animal care and usage and were conducted with approval from The University of Tokyo’s Animal Experiment Ethics Committee (approval number: P29-39 and P29-40), in accordance with the University of Tokyo’s guidelines for laboratory animal care and use. Adult male and female C57BL/6 J (B6) mice aged 5–8 weeks, along with E15 neonatal mice, were procured from SLC (Hamamatsu, Shizuoka, Japan). BTBR mice, aged 5–8 weeks, were sourced from the Jackson Laboratory (Bar Harbor, ME). E15 neonatal BTBR mice were generated through in-house breeding. Mice were housed under a 12/12 h light/dark cycle starting at 8 a.m., with access to food and water *ad libitum*. Temperature was maintained at 22°C–25°C, and humidity ranged from 40% to 65%.

### Drug administration

Mice were weighed and intraperitoneally injected in the abdominal area with 10 mg/kg vortioxetine hydrobromide (Combi-Blocks, San Diego, CA). Initially, the compound was dissolved in 100% dimethyl sulfoxide (DMSO) and subsequently diluted to a 1% DMSO concentration using water. Vehicle controls utilized 1% DMSO in water. In the dendritic spine analysis experiment involving fixed brain slices, vortioxetine or vehicle was administered once daily for seven doses until the day prior to fixation. In behavioral evaluations, vortioxetine or vehicle was administered once daily for seven doses until the day preceding the 3-chamber social interaction test. Moreover, after the 3-chamber test, mice were administered 10 mg/kg vortioxetine or vehicle, followed by self-grooming test the subsequent day.

### Behavioral tests

All animals utilized in the behavioral studies were male.

#### 3-chamber social interaction test

In the initial session of the behavioral assessment, mice were placed in the central chamber and allowed to explore all three chambers (each measuring 170 mm in length × 250 mm in width × 250 mm in height) freely for 10 min while being recorded. Before proceeding to the subsequent session, a mouse was isolated in the central chamber. One chamber contained an empty cage cup as a novel object, while the other chamber contained a cage cup with unfamiliar mouse. During the following 10-min recording, mouse had the freedom to explore and interact with novel object or unfamiliar mouse. Recorded video footage of each session was analyzed, and the duration spent in each chamber was quantified.

The sociability index for each animal was calculated as: Sociability index = Novel mouse/(Novel mouse + Novel object): where “Novel mouse” is the time spent in chamber including unfamiliar mouse in a cage cup and “Novel object” is the time spent in chamber including an empty cage cup.

#### Self-grooming test

A mouse was placed within an empty clear box (80 mm in length × 95 mm in width × 120 mm in height). Spontaneous mouse behavior was recorded for 10 min from both the front and rear of the box, following a 50-min box habituation period. Self-grooming time was visually assessed based on video recordings. Grooming behavior was assessed following the syntactic grooming chain pattern identified in mice in a prior study (Kalueff et al., 2007).

### Dendritic spine morphology analysis

Dendritic spine analysis in brain slices of adult male mice aged 7–8 weeks was achieved through the transfection of neurons with fluorescent protein genes via *in utero* electroporation at embryonic day 15. Dendritic spine analysis in cultured neurons was achieved by transfection of fluorescent protein genes through electroporation before seeding the prepared neurons.

#### Plasmid vector construction

The EGFP-actin cDNA was engineered as a fusion gene, with EGFP tagged at the N-terminus of murine β-actin (GenBank: BAE30357.1) via a flexible linker (SGGGGSGGGGSGGGGS) to preserve the in-frame structure. The cDNA sequences encoding EGFP and EGFP-actin were inserted into a mammalian expression vector harboring the cytomegalovirus immediate early enhancer-chicken β-actin hybrid (CAG) promoter, the woodchuck hepatitis virus posttranscriptional regulatory element (WPRE), and the rabbit β-globin polyadenylation signal.

#### *In utero* electroporation

*In utero* electroporation was performed following a previously described protocol (Tabata and Nakajima, 2001) with minor adjustments. Anesthetized pregnant females underwent midline abdominal incisions under deep isoflurane inhalation anesthesia. Embryonic uterine horns were gently exposed by creating gaps between embryos using ring-forceps. Plasmid DNA solution in normal saline (1–1.5 µL) was injected into the lateral ventricle of mouse embryos using a mouth-controlled pipette system and a pulled-glass micropipette. Fast Green solution (0.1%) was incorporated into the plasmid solution at a 1:10 ratio to enable injection monitoring. Injected embryos were subjected to electronic pulses (Poring Pulse; 40 V, 50 ms duration, 960 ms interval, 2 times/Transfer Pulse; 5 V, 50 ms duration, 50 ms interval, 3°times) using an electroporator (NEPAGENE, Japan). Electroporation targeted the ventricular zone within the dorso-lateral cerebral cortex. The injected mice were returned to the abdominal cavity, and confirmation was obtained that they underwent normal development until reaching 7–8 weeks of age post-birth.

#### Brain slice preparation

*In utero* electroporation was performed on all fetal mice, but only males were subjected to Brain Slice preparation. Mice under isoflurane anesthesia were transcardially perfused with 4% paraformaldehyde in phosphate-buffered saline to fix the whole body. Fixed brains were extracted, embedded in Optimal Cutting Temperature compound, frozen, and 16 µm frozen sections were generated using a cryostat. Mounted on glass slides, these fixed brain slices were utilized for imaging experiments.

In the observation of dendrites, the area with the appropriate labeled cell density was selected in layer 2/3 of the primary somatosensory cortex and primary motor cortex within a total area of 2 mm, +1.1 mm to −0.9 mm with respect to Bregma. Brain sections to be used for observation were selected based on the shape of the ventricles, referring to Brain atlas (Paxinos and Franklin, 2019).

#### Primary neuron cultures and transfections

Mouse primary neurons and astrocytes from the cerebral cortex and hippocampus were prepared through a modified version of a previously reported procedure (Jones et al., 2011). In brief, primary astrocytes from B6 postnatal mice (P5) were cultured on flasks for 7 days with Advanced-DMEM (Thermo Fisher Scientific, Waltham, MA, United States) supplemented with 10% FBS (Thermo Fisher Scientific, Waltham, MA, United States), 1% GlutaMAX-I (Thermo Fisher Scientific, Waltham, MA, United States), and 1% Antibiotic-Antimycotic Mixed Stock Solution (AAM, Nacalai Tesque, Kyoto, Japan). Subsequently, they were seeded at a density of 1.7 × 10^3^ cells per well in a 96-well glass-bottom plate.

Primary neurons were prepared from cortex and hippocampus of E15 mouse embryos of B6 mice or BTBR mice using DNase and papain treatment. Immediately, the prepared neurons and EGFP/EGFP-actin plasmids were mixed in cuvettes with electrode, then electroporated with charged electrical pulses (Poring Pulse; 285 V, 0.6 ms duration, 50 ms interval, 2 times/Transfer Pulse; 20 V, 50 ms duration, 50 ms interval, 5 times) using an electroporator (NEPAGENE, Japan) and then seeded onto the astrocyte feeder layer in 96-well plates at a density of 2.5 × 10^3^ cells per well. This means that co-cultures of neurons derived from B6 mice and astrocytes derived from B6 mice, as well as co-cultures of neurons derived from BTBR mice and astrocytes derived from B6 mice, were prepared. The next day, 1 µM Cytosine β-D-arabinofuranoside was added to inhibit astrocyte proliferation, and cultures were maintained for an additional 28 days in serum-free Neurobasal medium containing 1% GlutaMAX-1, 1% AAM, and 2% B27 supplement (Thermo Fisher Scientific, Waltham, MA, United States).

#### Dendritic spine imaging and quantitative analysis

Fluorescent neuron images were acquired using the SpinSR super-resolution microscope (Olympus) equipped with a UPlanSApo objective lens (×100, N.A. 1.35, Olympus). MetaMorph software (Molecular Devices) was used for image file processing. In fixed brain slice imaging and *in vitro* dendritic spine phenotype exploration, the acquired image size was 1,024 × 1,024 pixels, corresponding to a 40 × 40 µm field of view in super-resolution microscopy mode. For phenotypic screening, the image size was 1,024 × 1,024 pixels, covering a 120 × 120 µm area in confocal microscopy mode. All images had their *z*-axis ranges adjusted for each neuron in increments of 0.2 µm. Maximal intensity projections were used for all figures presented.

Three-dimensional analysis of reconstructed neurons and morphological analysis of dendritic spines were conducted using Neurolucida360 and NeuroExplorer software (MBF Bioscience). Initial dendrite tracing was performed manually with assistance from the Neurolucida360 system. Subsequently, Neurolucida360 automatically detected and classified dendritic spines based on predefined criteria (Dickstein et al., 2016; Rodriguez et al., 2008). In brief, spine detection is defined by the outer range from the center of the dendrite, the minimum height of the spine, detector sensitivity and minimum count to remove noise signals. The classification of spines is initially determined based on the head-to-neck ratio, where a head-to-neck ratio greater than 1.1 is categorized as “mushroom”, and a head-neck ratio less than 1.1 is designated as “stubby”. Within the “mushroom” classification, a head diameter exceeding 0.35 µm is considered as “mushroom”, while a head diameter not exceeding 0.35 µm is redirected to a “filopodium” determination. In the context of the “stubby” classification, a species is deemed suitable if the ratio of spine length to head diameter does not exceed 3. Conversely, if this ratio exceeds 3 (i.e., it is elongated), it is reassigned to a “filopodium” determination. “Filopodium” determinations are predicated on the total length of the spine, and are classified as “filopodium” if the length is 3 µm or more; otherwise, they are designated as “thin.”

The number of spines analyzed in each experiment is summarized in the Supplementary Table 1.

### *In vitro* electrophysiology

Spontaneous tetrodotoxin-resistant miniature excitatory postsynaptic currents (mEPSCs) were recorded using the whole-cell configuration of the patch clamp technique (Hamill et al., 1981). Borosilicate glass electrodes (Sutter Instruments) were pulled on a Flaming/Brown micropipette P-97 puller (Sutter Instruments) and fire-polished to achieve electrical resistances of 3–5 MΩ. All neuronal events were analyzed in 18–22°days *in vitro* (DIV) primary culture neurons by whole-cell patch-clamp recordings. EPC10 amplifier and Patchmaster software (HEKA Electronic) were used for voltage-clamp recordings. The internal solution contained (in mM) 130 K-gluconate, 2 NaCl, 20 HEPES, 4 MgCl_2_, 0.25 EGTA, 4 Mg-ATP, and 0.4 Tris-GTP, with pH adjusted to 7.4 using KOH. The bath solution contained (in mM): 130 NaCl, 2.5 KCl, 2 CaCl_2_, 1 MgCl_2_, 10 HEPES, and 10 glucose, with pH adjusted to 7.4 using NaOH. During spontaneous mEPSCs recordings, cell membrane potential was clamped at −60 mV in the presence of 1 µM tetrodotoxin in the bath solution. All responses were digitized at 10 kHz with a 5–1,000 Hz band-pass filter. A 3-min period was observed for mEPSCs, and series resistance was monitored before and after each recording. Recordings were conducted at room temperature, and miniAnalysis 6.0.3 software (Synaptosoft) was employed to detect mEPSC signals.

For some B6 mouse neurons, an internal solution containing 50 mM biocytin (Thermo Fisher Scientific, Waltham, MA, United States) was used. These cells were fixed after recording and fluorescently stained by streptavidin, Alexa Fluor 488 conjugate (Thermo Fisher Scientific, Waltham, MA, United States).

### Phenotypic screening

#### Specialized compound library

The compound library was procured from MedChemExpress (Princeton, NJ, United States) based on the following criteria: 1) diversity of target molecules, 2) central nervous system activity, and 3) confirmed safety through preclinical or clinical studies (MedChemExpress, Master of Bioactive Molecules; Inhibitors, Screening Libraries and Proteins, customed liblary). Refer to Table 1 for information on the diversity of target molecules and the compound count for each target molecule. A list of all compounds is provided in the Supplementary Table 2.

**TABLE 1:** List of hit compounds and target molecules. This table shows the number of compounds used for screening and the hit rate for each target molecule. “Candidate hit compounds” shows the results of the first assay performed at a single dose. “Hit compounds” indicated that “Candidate hit compounds” has passed the reproducibility test, including concentration dependence. “Hit rate” indicated the ratio of “No. of hit compounds” to “No. of compounds” for each target molecule. All compound names are mentioned for “Hit compounds”.

Target molecule	No. of compounds	No. of candidate hit compounds	No. of hit compounds	Hit rate (%)	Name of hit compounds
5-HT receptor	38	14	5	13.2	Vortioxetine
					Risperidone
					8-OH-DPAT
					Tandospirone
					Palonosetron
Adrenergic receptor	23	9	1	4.3	Indacaterol
Dopamine receptor	17	4	1	5.9	Rotigotine
mACh receptor	17	7	1	5.9	Diphenmanil
GABA receptor	11	5	1	9.1	AWD 131–138
γ-secretase	8	3	0	0	-
AMPA receptor	8	4	0	0	-
nACh receptor	8	3	2	25	A-867744
					Cisatracurium
LRRK2	8	4	1	12.5	HG-10–102-01
NMDA receptor	7	3	1	14.3	Mephenesin
Opioid receptor	7	3	1	14.3	JTC-801
Monoamine oxidase	6	1	0	0	-
Neurokinin receptor	6	3	1	16.7	Maropitant
FAAH	4	1	0	0	-
SSRI	4	0	0	0	-
Amyloid-β	3	0	0	0	-
AChE	2	0	0	0	-
CGRP receptor	2	1	0	0	-
β-secretase	1	1	0	0	-
MAGL	1	0	0	0	-
Total	181	66	15	8.3	

#### Compound treatment and image acquisition

In the first screening, compounds were treated at a final concentration of 1 µM on the 15 DIV. In the second screening, compounds were administered at doses tailored to each compound’s profile. Compounds with potent activity or high toxicity were tested were further tested at multiple, lower concentrations. In a secondary screening conducted to confirm reproducibility, the maximum concentration was set at 1 μM, with lower concentrations tested down to 0.01 µM in 2-fold (1, 0.5, 0.25, 0.13, 0.06, 0.03 μM) or 3-fold (1, 0.3, 0.1, 0.03, 0.01 μM) dilutions.

For each compound and treatment condition, twenty neuron images were captured. Neurons were selected by scanning the field of view in a defined trajectory, starting from the EGFP-labelled neuron closest to the central coordinates of the well. The first twenty EGFP-labelled neurons encountered, including those at the starting point, were systematically selected. Each neuron’s image covered a 120 × 120 µm field of view centered around the cell body, encompassing all dendrites within approximately a 60 µm radius from the cell body for assessment.

The toxicity of the compounds was assessed based on the presence or absence of morphological changes in individual neurons. Specifically, cells were deemed damaged due to compound toxicity if their cell bodies exhibited a rough texture with numerous fine irregularities in the contours and if broken neurites were observed, in fluorescent images under a microscope. Cells with findings of damage were annotated as “Damaged cells” and were not included in the determination of “Mature or Immature Spine Neurons.”

#### Hit compounds selection

All image files were anonymized, and a skilled assessor determined whether the percentage of mushroom-type spines in each neuron was more or less than 50%. Neurons with over 50% mushroom-type spines were categorized as “Mature Spine Neurons”, while those with less than 50% mushroom-type spines were designated as “Immature Spine Neurons”. For each image treated with a compound and DMSO, the percentage of “Mature Spine Neurons” among 20 images was calculated. Before starting the screening we confirmed that B6 mouse neurons exhibited a 10% higher proportion of “Mature Spine Neurons” compared to BTBR mouse neurons. Therefore, compounds that increased the percentage of mature spine neurons by more than 10% compared to DMSO treatment were identified as hit candidate or hit compounds. For all assays, DMSO-treated groups were prepared for two wells, and the average of the two wells was used as the reference value for hit criteria. Compounds that were excluded due to toxicity in the initial screening were to be re-assayed at lower treatment concentrations, taking into considering their activity values; compounds that exceeded the DMSO-treated group by 10% were designated for a second evaluation. In the second evaluation, the maximum concentration was set at 1 μM, with concentrations set at 2- or 3-fold lower concentration.

### Statistical analysis

Statistical analyses employed the Kruskal–Wallis test with Dunn’s *post hoc* test and Mann-Whitney *U* test. GraphPad Prism 6 software (GraphPad, San Diego, California, United States) was utilized for all statistical analyses. Statistical significance was set at *p* < 0.05 and was represented as *p < 0.05 or **p < 0.01. All presented results were expressed as mean ± standard error of the mean (SEM). Detailed statistical information and the number of animals used are provided in the figure legends.

## Results

### Dendritic spine phenotype identification in the BTBR mouse

**FIGURE 1:**
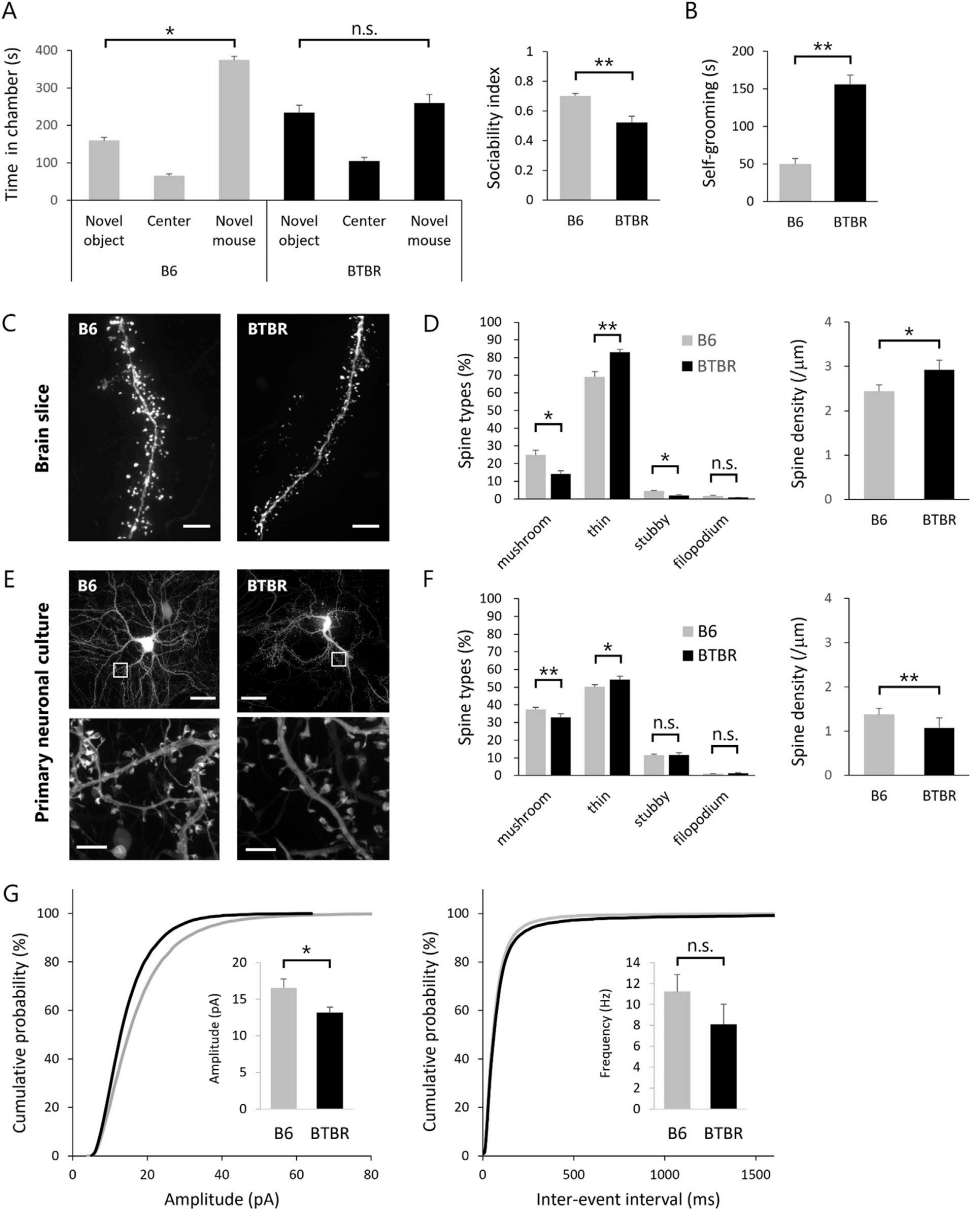
Replication of abnormal spine morphology in BTBR mice with *in vitro* cultured neurons **(A,B)** Validation of ASD-like behavior in BTBR mice. **(A)** Comparison of social behavior between B6 (n = 13) and BTBR (n = 16) mice using the 3-chamber social interaction test. Left graph shows time spent in each chamber. Right graph sociability index. **(B)** Duration of time spent in grooming behavior per 10 min. **(C,D)** Quantitative analysis of dendritic spine phenotype in BTBR mice. **(C)** Images of dendrite and spines. Scale bar is 20 μm. **(D)** Comparison of spine types and comparison of spine density. **(E,F)** Quantitative analysis of dendritic spine phenotype in primary neuronal culture from BTBR mice. **(E)** Images of cultured neurons (top) and dendritic spines (bottom). Scale bar is 20 μm in top and 2 μm in bottom, the enlarged section. **(F)** Comparison of spine types and comparison of spine density. **(G)** Normalized cumulative distribution analysis of mEPSC amplitude and frequency and inset of averaged mEPSC amplitude and frequency. All bar graph data represent mean ± SEM, *p < 0.05, **p < 0.01, Kruskal–Wallis test with Dunn’s *post hoc* test [Left panel in **(A)**] and Mann-Whitney *U* test (Other statistical tests except Left panel in **(A)**).

ASD model mice are expected to exhibit ASD-like behavioral traits. In this study, we validated the manifestation of ASD-like behavior in BTBR mice through the assessment of abnormal social behavior and repetitive behavior (Figures 1A, B). Abnormal social behavior of BTBR mice was assessed using the sociability test of the 3-chamber social interaction test. In an early paper that identified BTBR mice as a mouse model for ASD (Moy et al., 2007), a robust phenotype was found in the sociability test, while no impairment was found in the social novelty test. Although some subsequent studies have reported the detection of social novelty test deficits in BTBR mice, we employed the sociability test as a more robust assessment and evaluated core symptoms of ASD, such as lack of social interest and avoidance of social contact. Repetitive behavior was assessed by quantifying self-grooming. The self-grooming test was chosen as a more robust assessment because there are a lot of studies for drug efficacy using self-grooming test with BTBR mice (Meyza and Blanchard, 2017). As reported, BTBR mice displayed reduced interaction time with unfamiliar mice (Figure 1A) and increased self-grooming behavior duration per unit of time (Figure 1B) in comparison to B6 mice.

Subsequently, we explored phenotypic variations in cortical excitatory neurons of BTBR mice. Our phenotypic search encompassed two distinct fluorescent protein genes with distinct expression modes, namely, cytoplasmically expressed EGFP (EGFP) and actin-fused EGFP (EGFP-actin), introduced into neurons within the cortical somatosensory cortex of embryonic mice using *in utero* electroporation. EGFP expression in the cytoplasm facilitates the visualization of the cell body and dendrites but proves inadequate for the visualization of the spine, particularly in its immature morphology. Given that spines are constructed through a highly polymerized actin cytoskeleton, the introduction of EGFP-actin, wherein the EGFP protein is expressed downstream of the actin molecule, allowed comprehensive visualization of the entire spine structure. Using super-resolution microscopy, fluorescence images of excitatory neurons were obtained from fixed brain slices of EGFP and EGFP-actin-transfected B6 and BTBR mice (Figure 1C). For precise morphological analysis, uniform labeling of dendrites, spine head, and spine neck with equivalent fluorescence intensity is crucial. While EGFP labeling was suitable for dendrites, EGFP-actin was more suitable for spine head and neck labeling. This study employed both EGFP and EGFP-actin for labeling, ensuring equivalent fluorescence intensity observation of spines and dendrites. Acquiring images in three dimensions, we quantified the morphology of individual spines, classified into mushroom, thin, stubby, and filopodium types based on morphological characteristics (Hering and Sheng, 2001). The spines of B6 and BTBR mice were classified into four categories based on quantitative analysis, revealing that BTBR mice exhibited a significantly lower proportion of mature spines (mushroom type) and a significantly higher proportion of immature spines (thin type) compared to B6 mice (Figure 1D).

### *In vitro* dendritic spine phenotypes

The primary objective of this study is to identify compounds that target the core symptoms of ASD based on the spine phenotype. Therefore, an *in vitro* primary neuron culture system was established to mimic the BTBR mouse spine phenotype. Primary cultured neurons transfected with EGFP and EGFP-actin were observed by super-resolution microscopy at 21 DIV for quantitative analysis of spines as in the case of brain slices (Figure 1E). Neurons cultured from BTBR mice showed a statistically significantly lower proportion of mature spines (mushroom type) and a higher proportion of immature spines (thin type) compared to B6 mice. The spine density of BTBR mouse neurons *in vitro* was also lower than that of B6 mouse neurons (Figure 1F). In addition, dendritic spine classification and ratios were consistent between brain slices and cultured neurons, indicating that the BTBR mouse spine phenotype was faithfully represented in cultured neurons.

Given that dendritic spines are postsynaptic structures, alterations in spine morphology could impact postsynaptic function. Thus, mEPSC, indicative of postsynaptic function, was measured in neurons from B6 and BTBR mice within the same culture period as spine morphology observation. The results showed a reduction in mEPSC amplitude in BTBR mouse neurons compared to B6 mouse neurons, consistent with the higher proportion of morphologically immature spines in BTBR mice (Figure 1G).

Biocytin was applied to neurons from B6 mice during mEPSC measurement, and the spine morphology of neurons without EGFP and EGFP-actin was visualized by fluorescent staining with streptavidin-GFP after the measurement (Supplementary Figure 1). The spine morphology closely resembled that of EGFP- and EGFP-actin-transfected neurons, indicating that transfection of EGFP and EGFP-actin does not alter spine morphology. The most important point is that both methods were able to visualize and quantitatively evaluate not only large spines such as mushroom type but also extremely fine structures such as thin and filopodium type. In addition, previous studies have shown that actin-GFP does not affect neuronal morphology (Iqnacz et al., 2023).

### Phenotypic assay for compound screening

**FIGURE 2:**
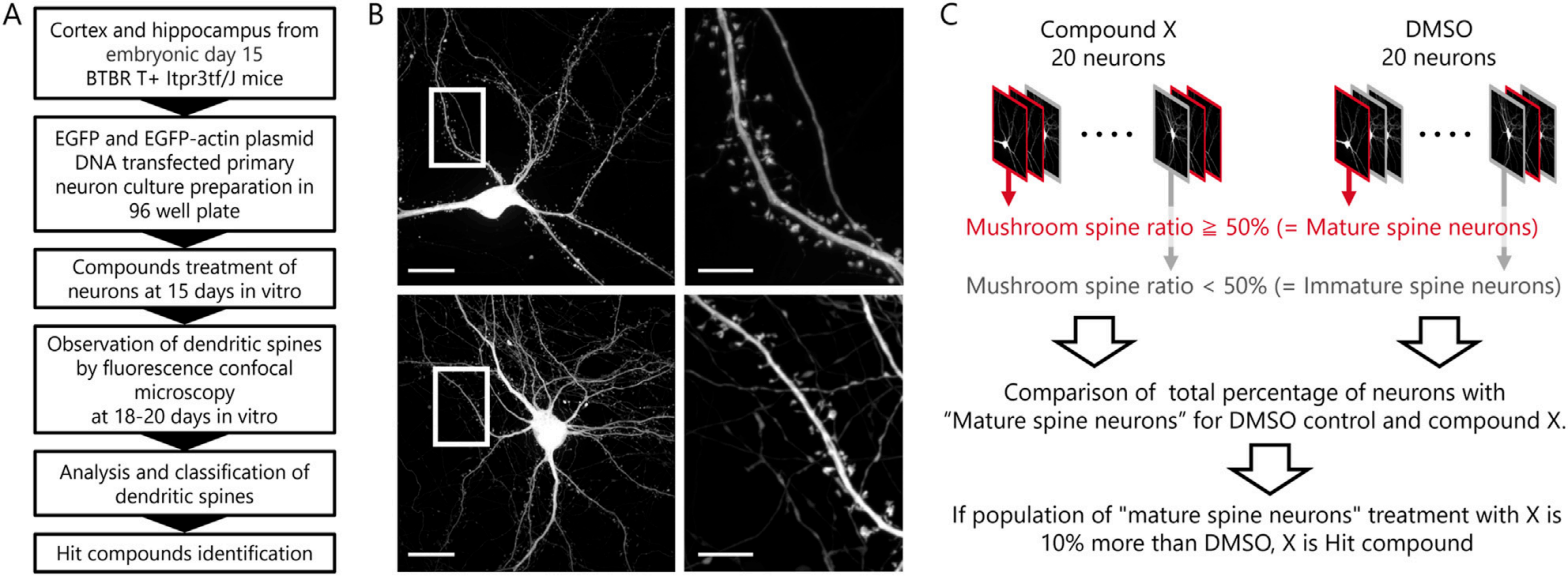
Phenotypic screening scheme and hit compound criteria definition. **(A)** Scheme from preparation of primary cultured neurons to assay of compounds. **(B)** Example images of a compound-treated cultured neuron in top row and a DMSO-treated cultured neuron in bottom row (Scale bar; 20 μm). The white framed area is enlarged in the right images (Scale bar; 5 μm). **(C)** Schematic diagram of the procedures and definitions for determining hit compounds.

Subsequent to *in vitro* observations, a compound screening procedure was executed using *in vitro* neuronal cultures from BTBR mice (Figure 2A). A library comprising 181 compounds with established activity for phenotypic assays was screened. At 15 DIV, each well of a 96-well plate received either DMSO or compound treatment. Fluorescent images of 20 neurons per well were obtained during the 18 to 20 DIV period (Figure 2B). Analysis of dendritic spines was carried out, categorizing neurons with over 50% mushroom-type spines as “mature spine neurons” and those with less than 50% mushroom-type spines as “immature spine neurons” (Figure 2C). Before starting the screening, it was comparing B6 and BTBR mouse neurons regarding the percentage of mushroom-type spines, the proportion of “Mature Spine Neurons” in B6 mice was approximately 10% higher than in BTBR mice [B6 mouse neurons: 35.5% ± 6.7%, BTBR mouse neurons: 24.7% ± 5.4% (mean ± SEM)]. Considering the criteria that hit compounds should ameliorate the phenotype of BTBR mouse neurons to match that of B6 mouse neurons, compounds exceeding the DMSO percentage by over 10% were identified as hit candidate or hit compounds in the screening.

The initial screening was performed at a single concentration of 1 μM. Although re-assays at lower concentrations were planned for compounds for which 20 cells could not be selected due to cytotoxicity, no compounds actually had to be re-assayed due to toxicity. Of the 181 compounds evaluated, 66 were identified as candidate hit compounds in the initial screening. In the secondary screening, reproducibility was ascertained at concentrations deemed optimal, as determined by the 1 μM standard. Of the 66 compounds subjected to this evaluation, 15 were identified as hit compounds (hit rate: 8.3%, Table 1; Supplementary Table 3).

Among the 15 hit compounds, some exhibited multiple hits on the same target molecule, rendering them highly probable targets. Specifically, five compounds targeted the 5-hydroxytryptamine receptor (5-HTR), and two targeted the nicotinic acetylcholine receptor (nAChR). Activities of compounds targeting 5-HTR varied, including selective 5-HT_1A_R agonists (8-OH-DPAT and Tandospirone), 5-HT_3_R antagonist (Palonosetron), and compounds with combined activities against 5-HT_1A_R agonist and 5-HT_3_R antagonist (Vortioxetine). Notably, Vortioxetine also has 5-HT_7_R antagonist and serotonin selective reuptake transporter (SERT) inhibitor activity. Risperidone is classified as a 5-HTR target compound because it has 5-HT_2_R antagonist activity, but it also has dopamine D2 receptor antagonist activity.

For nAChRs, A-867744 is a selective α7 nAChR agonist and has activity on nAChRs expressed in the nervous system, whereas Cisatracurium is a nAChR antagonist and has activity on nAChRs expressed in muscle.

### *In vivo* efficacy of hit compounds on spine phenotype

In the *in vivo* experiments, the more promising compounds were selected from the seven hits in the two groups with common targets. The two compounds targeting the nAChR had different mechanisms of action on the nAChR, while some compounds targeting the 5-HTR subtype had common mechanism of action on the 5-HTR. Therefore, we hypothesized that compounds targeting the 5-HTR subtype were more likely to promote spine maturation *in vivo*. First, risperidone was excluded because it is well known to have no effect on the core symptoms of ASD. The remaining four compounds were 8-OH-DPAT and tandospirone, which are 5-HT_1A_R agonists; palonosetron, a 5-HT_3_R antagonist; and vortioxetine, which has both 5-HT_1A_R agonist and 5-HT_3_R antagonist activity. Based on the above results, since 5-HT_1A_R agonist and 5-HT_3_R antagonist activities are considered important for spine maturation, vortioxetine, which has both activities, was evaluated as the highest priority compound. We set the dose at which vortioxetine is expected to occupy a certain percentage of 5-HT_1A_R and 5-HT_3_R in the *in vivo* administration experiments. Based on previous studies of vortioxetine doses and the occupancy of various 5-HTR subtypes in the rat brain (Sanchez et al., 2015), the dose for BTBR mice was set at 10 mg/kg.

**FIGURE 3:**
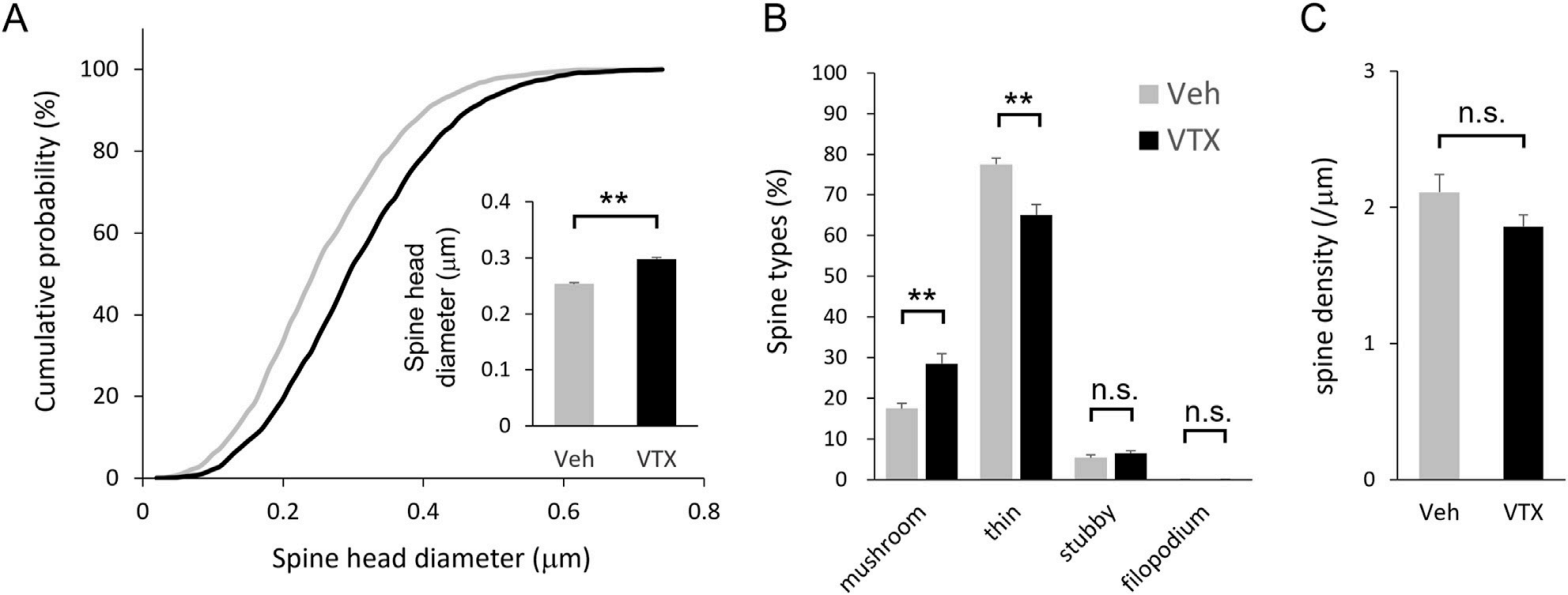
Efficacy of vortioxetine on *in vivo* spine phenotypes of BTBR mice. Comparison between vehicle (“Veh”; 1% DMSO) and vortioxetine (“VTX”) administration based on spine analysis using fixed brain slice. Doses were administered at 10 mg/kg once daily for 7 days. **(A)** Normalized cumulative distribution analysis of dendritic spine head diameter and inset of averaged dendritic spine head diameter. **(B)** Comparison of spine morphology based on four spine type classifications. **(C)** Comparison of spine density based on total number of spines. All bar graph data represent mean ± SEM, *p < 0.05, **p < 0.01, Mann-Whitney *U* test.

Following transfection of EGFP and EGFP-actin in the embryonic period, brain slices were prepared from adult BTBR mice for spine morphology analysis. Notably, vortioxetine treatment for 7 days led to larger spine head diameters and a shift towards larger spine head sizes (Figure 3A). Morphological classification analysis showed increased proportions of mushroom-type spines and decreased proportions of thin-type spines (Figure 3B). However, no significant differences in spine density were observed between DMSO- and vortioxetine-treated groups (Figure 3C), indicating that vortioxetine promotes maturation rather than density change in BTBR mouse spines.

### Efficacy of vortioxetine on ASD-like behavior in BTBR mice

**FIGURE 4:**
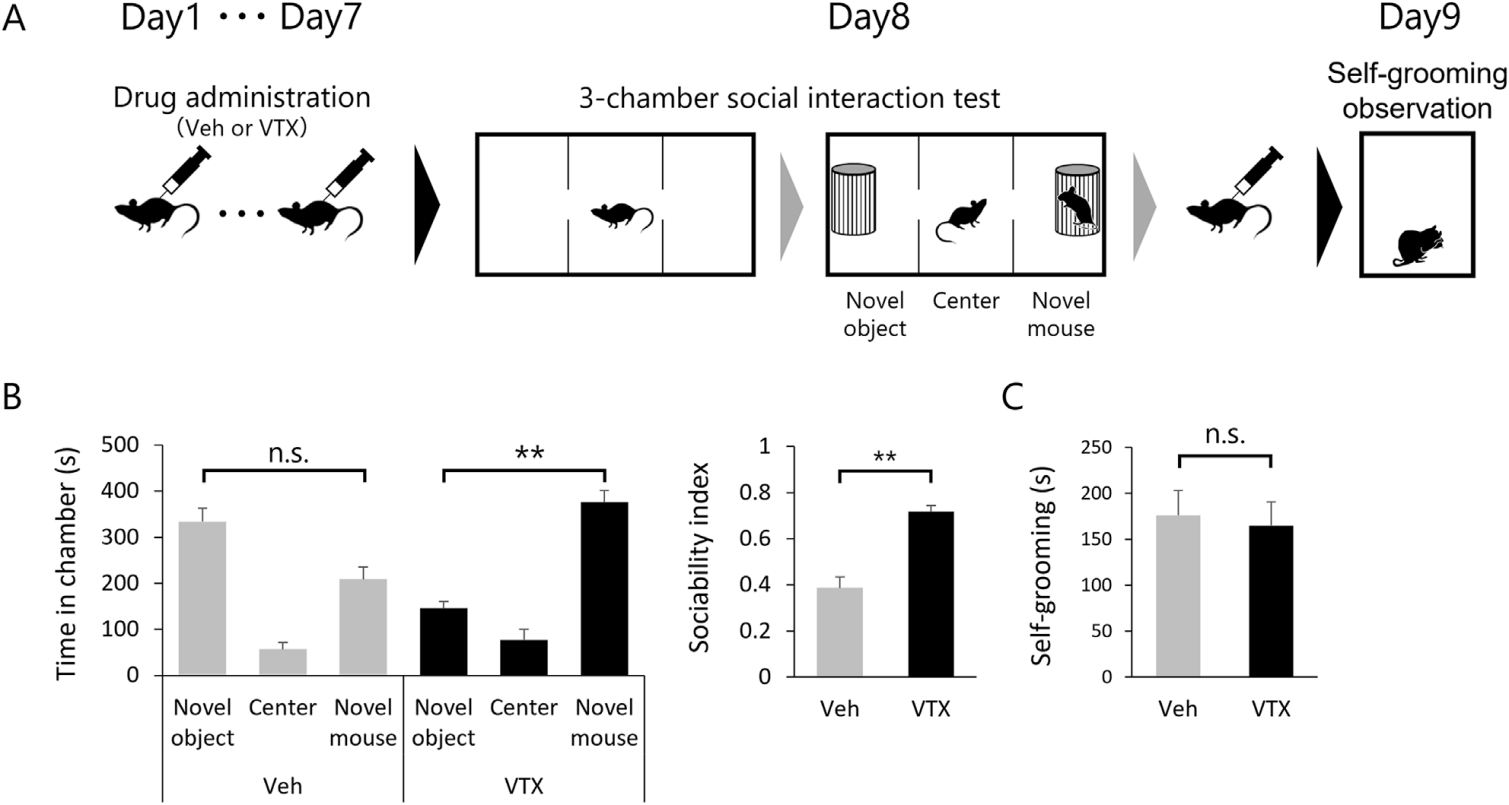
Efficacy of vortioxetine on ASD-like behavior in BTBR mice. **(A)** Scheme of behavioral testing in mice treated with vehicle (“Veh”; 1% DMSO, n = 10) or vortioxetine (“VTX”, n = 12). Dosing of 10 mg/kg vortioxetine once daily for 7 days up to 24 h before the 3-chamber test and followed by administration after the 3-chamber test again, and self-grooming was assessed the day after. **(B)** Comparison of social behavior using the 3-chamber social interaction test. Left graph shows time spent in each chamber. Right graph sociability index. **(C)** Duration of time spent in grooming behavior per 10 min. All bar graph data represent mean ± SEM, *p < 0.05, **p < 0.01, Kruskal–Wallis test with Dunn’s *post hoc* test [Left panel in **(B)**] and Mann-Whitney *U* test [Right panel in **(B)** and **(C)**].

The favorable impact of 7-day vortioxetine treatment on spine phenotype in adult BTBR mice led to investigations into its potential effects on ASD-like behavior (Figure 4A). Sociability was evaluated through a 3-chamber social interaction test, while repetitive behavior was assessed by self-grooming test. The DMSO-treated group tended to have a shorter interaction time with the novel mouse compared to the interaction time with the novel object, whereas the vortioxetine-treated group had a statistically significantly longer interaction time with the novel mouse. The sociability index also indicated significantly higher values in the vortioxetine-treated group (Figure 4B). However, self-grooming behavior did not show significant differences between the two groups (Figure 4C), suggesting that vortioxetine ameliorates social impairment but not repetitive behavior in BTBR mice.

## Discussion

This study centered on the dendritic spine phenotype of model mice exhibiting ASD-like behavior and identified compounds that improve spine phenotype and ASD-like behavior.

Initially, a compound screening system founded on spine phenotypes was established. A spine phenotype was identified in the cortex of BTBR mice with ASD-like behavior independent of specific genetic mutations. It was verified that this phenotype can be replicated in primary cultured neurons from BTBR mice.

A compilation of 181 compounds, sourced from well-established pharmaceuticals with documented effects on the central nervous system and established safety profiles, was curated into a specialized library intended for phenotypic screening. Subsequent screening procedures yielded 15 compounds that fulfilled the designated criteria, thus emerging as potential hits.

Subsequently, the analysis of the hit compound group unveiled suitable combinations of 5-HTR subtypes. Our attention was directed towards vortioxetine, revealing its capability to improve the spine phenotype of BTBR mice along with their social impairment.

### The utility of screening systems based on spine phenotype

Given that spine morphology anomalies are implicated in ASD and genetic disorders featuring ASD-like symptoms, the hypothesis posits that spine phenotypes are pivotal in manifesting the core ASD symptoms. As spine dynamics are influenced by interactions among various neuronal signal inputs and their downstream molecules, pinpointing compounds affecting spine abnormalities through single-target-based compound screening presents challenges. Consequently, this study adopts a phenotype-based rather than a target-based approach.

The primary advantage of phenotypic screening is its potential to yield compounds characterized by multiple target activities, which may be more potent than compounds designed to target a single molecule. Given these premises, employing model animals or cells rooted in specific genetic mutations was deemed unsuitable for phenotypic assays. However, the use of naïve cells, such as neurons from B6 mice, is also not suitable for the purposes of this study. The mechanism may be fundamentally different between the action in further maturing the normal spine structure of naive neurons and the action in model animals to mature the immature spines to normal levels. Therefore, we focused on obtaining compounds that could improve the phenotype of the pathological model rather than obtaining compounds that simply increase or enlarge spines. This required the use of neurons from ASD models with abnormal spine morphology, such as BTBR mice.

This study, for the first time, substantiates aberrant spine morphology in the cortex of BTBR mice (Figures 1C, D). Furthermore, we established that a similar phenotype can be replicated in primary cultured neurons derived from the cortex of BTBR mouse embryos (Figures 1E, F).

The pursuit of *in vitro* phenotypic representation centered on two pivotal issues. Firstly, the conditions for fluorescent protein labeling were optimized to enable consistent observation of neuronal structures from the macroscopic to the microscopic level. This was achieved through the transfection of two types of EGFP proteins, cytoplasmically expressed EGFP and actin fused EGFP. The former labeled soma and dendrites with heightened fluorescence intensity, while the latter marked the microstructure of spines containing densely polymerized actin. The second concern revolved around establishing conditions conducive to culturing neurons with mature functions akin to those *in vivo*. Regarding the latter concern, culture conditions and the timing of spine observation were assessed using neurons from B6 mice. Co-culturing with astrocytes proved indispensable for cultivating neurons with mature spines. Notably, even under co-culture conditions, the number and length of neurites were still limited at around 7 DIV, mostly with low-density immature spines. Subsequently, at 14–21 DIV, neurites displayed increased length, branching, spine density, and predominantly mature spine morphology. Electrophysiological analysis confirmed functional maturation through constant frequency and amplitude measurements of mEPSCs during this culture period (Figure 1G). In relation to the co-culture with astrocytes, it is notable that in this study, neurons from BTBR mice were co-cultured with astrocytes from B6 mice. The goal of this study was to search for compounds that could act directly on neurons and improve their ASD phenotype. Co-culture of astrocytes with neurons from BTBR mice would be superior in terms of *in vivo* reproduction but may not be able to extract the ASD phenotype as a property of the neurons themselves. In other words, it may be impossible to distinguish whether the phenotype of a neuron reproduced *in vitro* is caused by the neuron or a cell other than the neuron. In Figure 1, the difference in the phenotype of spine density in BTBR mice may be due to differences in non-neuronal cellular conditions.

While spine density results differed between brain slices and cultured neurons, this disparity does not affect our study’s objectives. The key endpoint here is the percentage of mature spines per neuron. It’s crucial that the mushroom-type to thin-type spine ratio observed in BTBR mouse brain slices can also be reproduced in cultured neurons. A possible reason for the lack of replication of *in vivo* spine density *in vitro* may be differences in neuronal density. Owing to the technical limitations of planar culture, the cell density of cultured neurons is substantially diminished when compared to the *in vivo* milieu, where neurons are distributed in three dimensions. This reduction in cell density *in vitro* consequently results in a decreased relative number of synaptic inputs per cell, thereby accounting for the observed lower spine density.

Finally, it should be noted that a hit compound identified in an *in vitro* phenotypic screening may not necessarily function as a therapeutic agent. For instance, risperidone, identified as a hit compound in this screening, failed to enhance performance in the 3-chamber test (Supplementary Figure 2A). Furthermore, it is widely recognized that risperidone does not alleviate core symptoms in ASD patients in clinical practice. While phenotypic screening serves as an effective tool in the early stages of drug discovery for narrowing down compounds, its potential as a therapeutic agent must be validated through *in vivo* testing. Thus, it is imperative to adopt a comprehensive approach, similar to the one employed in this study, which involves hit compound identification through screening, refinement of *in vivo* phenotyping, and improvement of behavioral assessments.

### Maturation of spines by compounds identified from phenotypic screening

Screening predicated on spine phenotype identified 15 hit compounds from a 181-compound library (Figure 2A). While the initial library contained 38 compounds targeting the 5-HTR (about 21% of the library), 5 out of 15 were directed at the 5-HTR (Table 1). These five compounds exhibited both shared and distinct functional profiles regarding the 5-HTR subtype. Specifically, 8-OH-DPAT, Tandospirone, and Vortioxetine shared agonist activity against 5-HT_1A_R. Similarly, Palonosetron and Vortioxetine featured antagonist activity against 5-HT_3_R. Risperidone, however, exhibited 5-HT_2_R antagonist activity, differing from other compounds.

Regarding mechanisms promoting spine maturation, the 5-HT_1A_R agonist potentially inhibits cytoskeletal degradation via GSK3β inactivation (Hajka et al., 2021; Polter and Li, 2011). Immature spines hover in an unstable state between loss and maturation. As actin-rich microstructures, spines are affected by GSK3β inactivation, possibly inhibiting cytoskeletal degradation and promoting transformation into mature forms characterized by stable dynamics. Spine maturation effects were also observed with the 5-HT_3_R antagonist. The 5-HT_3_R is distinctive among the seven 5-HTR subtypes in that it is the only ion channel-type receptor. Nonetheless, there are no reported instances of alterations in spine dynamics resulting from the antagonism of this channel, and the specific mechanism responsible for spine maturation remains unclear. Similarly, the role of 5-HT_2_R antagonists in spine maturation remains unclear.

It should also be noted that two compounds targeting the nAChR were found from screening. In particular, the α7 nAChR is known to be implicated in the pathophysiology of ASD and is a promising therapeutic target (Deutsch et al., 2015). Previous research has documented that α7 nAChR positive allosteric modulator has been shown to ameliorate abnormal social behavior in a study involving BTBR mice (Gee et al., 2014). These results substantiate the reliability of our screening system, which identified the α7 nAChR agonist as a potential hit compound.

### Factors underlying the improvement of ASD-like behavior by vortioxetine

In the present study, repeated administration of vortioxetine improved the spine morphological phenotype of cortex neurons in a mouse model of autism, while simultaneously ameliorating core symptoms of ASD. It should be noted, however, that these results do not indicate a causal relationship between improvement in spine phenotype and improvement in core symptoms.

The potential for vortioxetine to influence spine morphology in animals has been previously demonstrated in the rat hippocampal slice culture experiments (Waller et al., 2016). Waller et al. showed that treatment with 0.5 μM vortioxetine enlarged the spines of cultured hippocampal slice neurons from normal rats. However, there are no reports indicating that vortioxetine administration to pathological rodent models improves their spine phenotype. The effects of vortioxetine single administration on social behavior have also been documented in normal rats and BTBR mice. In experiments with normal rats, single doses of vortioxetine ranging from 0.25 to 8.0 mg/kg have been shown to increase social behavior under dosing conditions of 2.0 mg/kg and higher (Mørk et al., 2011). On the other hand, a study in which BTBR mice received a single dose of 5 mg/kg or 10 mg/kg of vortioxetine did not demonstrate complete improvement in social behavioral deficits (Witt et al., 2019). In fact, our single-dose experiments did not enhance the performance of BTBR mice in the 3-chamber social interaction test (Supplementary Figure 2B). Based on the results of these previous studies and the validation with single administration, we conducted a validation with repeated administration and demonstrated its efficacy. Studies on repeated dosing of vortioxetine have reported the following: chronic intake of vortioxetine via drinking induced an upregulation in gene expression associated with synaptic plasticity and ameliorated cognitive dysfunction in a mouse model of age-related cognitive decline (Waller et al., 2017). These studies share two common points: repeated administration of vortioxetine improved aspects related to synaptic function and behavioral deficits in pathological animal models.

Apart from its direct effects on neurons, such as 5-HT_1A_R agonist action and maturation of spine morphology, vortioxetine could address chronic neural signaling abnormalities in ASD, notably serotonin signaling. Elevated peripheral serotonin levels in ASD imply reduced central nervous system and synaptic cleft serotonin levels (Müller et al., 2016). Vortioxetine’s SERT inhibitory activity could augment synaptic cleft serotonin levels. Animal studies further reveal vortioxetine administration significantly elevates serotonin levels in the mPFC and hippocampus, driven not just by SERT inhibition, but by interaction between SERT inhibition and 5-HT_3_R antagonist activity, underscoring vortioxetine’s multi-targeting versatility (Mørk et al., 2011). However, being multi-targeted may not be advantageous in all aspects as a therapeutic agent for ASD. In this study, chronic administration of vortioxetine failed to improve the repetitive behavior of BTBR mice. Previous studies have shown that 5-HT_1B_R agonists elicit repetitive behavior in rodents and humans. On the other hand, SSRIs such as fluoxetine can prevent 5-HT_1B_R agonist-induced repetitive behavior. The fact that vortioxetine contains multiple, conflicting mechanisms of activity on repetitive behavior might be a factor in its limited effect on repetitive behavior.

The limitation of this study is that only BTBR mice were used for *in vivo* evaluation. Although the BTBR mouse is useful as a model of idiopathic ASD, it should be noted that the BTBR mouse does not cover the full range of ASD pathologies and phenotypes. Although we have only used BTBR mice in this study from an early stage drug screening perspective, evaluation of *in vivo* efficacy using multiple disease models is essential to predict clinical efficacy of drug candidates.

In summary, we proposed that dendritic spine phenotypes are important in ASD drug discovery, and we successfully confirmed spine abnormalities in BTBR mice, which exhibit ASD-like behavior independent of specific genetic mutations, and established an *in vitro* phenotypic screening system. Vortioxetine obtained in this screening system ameliorated spine abnormalities and social behavioral abnormalities, but not repetitive behavior, *in vivo*. These results demonstrate the potential of vortioxetine for the treatment of ASD and the usefulness of this screening system in ASD drug discovery.

## Data Availability

The raw data supporting the conclusions of this article will be made available by the authors, without undue reservation.
