# Supplementary material for: Development of an in vitro compound screening system that replicate the in vivo spine phenotype of idiopathic ASD model mice
Source: Front Pharmacol. 2024 Aug 29;15:1455812. doi: 10.3389/fphar.2024.1455812 (PMC11403255; doi:10.3389/fphar.2024.1455812)
Supplement: Supplementary file 1 [file DataSheet1.pdf]

Supplementary tables

Supplementary table 1. The number of spines analyzed in each experiment.

Figure 1D	Fixed brain slice	B6	3436 spines from 25 dendrites of 3 mice
		BTBR	1787 spines from 11 dendrites of 3 mice
Figure 1F	Primary neuronal culture	B6	7544 spines from 63 dendrites of 20 neurons
		BTBR	5767 spines from 65 dendrites of 20 neurons
Figure 3	Fixed brain slice	Vehicle	1570 spines from 17 dendrites of 3 mice
		Vortioxetine	1937 spines from 20 dendrites of 3 mice

Supplementary table 2. List of all compounds for phenotypic screening.

Target molecule	Compound name	CAS No.
5-Hydroxytryptamine (5-HT) receptor	Ferulic acid (sodium)	24276-84-4
	Azasetron (hydrochloride)	123040-16-4
	Rupatadine (Fumarate)	182349-12-8
	LY310762	192927-92-7
	Pimavanserin	706779-91-1
	Eletriptan (hydrobromide)	177834-92-3
	Granisetron (Hydrochloride)	107007-99-8
	Vortioxetine	508233-74-7
	CP-809101 (hydrochloride)	1215721-40-6
	Cisapride	81098-60-4
	Tandospirone	87760-53-0
	Setiptiline	57262-94-9
	Sarpogrelate (hydrochloride)	135159-51-2
	RS 127445	199864-87-4
	Flibanserin	167933-07-5
	SB-742457	607742-69-8
	Loxapine	1977-10-2
	LY 344864	186544-26-3
	Aripiprazole	129722-12-9
	Zolmitriptan	139264-17-8
	Prucalopride (succinate)	179474-85-2
	BRL 54443	57477-39-1
	Clozapine (N-oxide)	34233-69-7
	Mosapride (citrate)	112885-42-4
	Ramosetron (Hydrochloride)	132907-72-3
	Piboserod	152811-62-6
	Tropisetron (Hydrochloride)	105826-92-4
	Rizatriptan (benzoate)	145202-66-0
	Dalasetron (Mesylate hydrate)	878143-33-0
	Palonosetron (Hydrochloride)	135729-62-3
	Ondansetron	99614-02-5
	Tianeptine (sodium salt)	30123-17-2
	Mirtazapine	85650-52-8
	SB 242084	181632-25-7
	Adoprazine	222551-17-9

	TG6-10-1	1415716-58-3
	Risperidone	106266-06-2
	8-OH-DPAT	78950-78-4
Adrenergic receptor	Silodosin	160970-54-7
	Deoxycorticosterone (acetate)	56-47-3
	Indacaterol (maleate)	753498-25-8
	Nebivolol (hydrochloride)	152520-56-4
	Phentolamine (mesylate)	65-28-1
	Scopine (hydrochloride)	85700-55-6
	ICI 118,551 (hydrochloride)	72795-01-8
	(S)-Timolol (Maleate)	26921-17-5
	Esmolol (hydrochloride)	81161-17-3
	Methoxyphenamine (Hydrochloride)	5588-10-3
	Atipamezole (hydrochloride)	104075-48-1
	Levobetaxolol (hydrochloride)	116209-55-3
	Ritodrine (hydrochloride)	23239-51-2
	Carvedilol	72956-09-3
	Tamsulosin	106133-20-4
	Tizanidine (hydrochloride)	64461-82-1
	Formoterol (Fumarate)	43229-80-7
	Salmeterol (xinafoate)	94749-08-3
	Ivabradine (hydrochloride)	148849-67-6
	Naftopidil	57149-07-2
	UK 14,304 (tartrate)	70359-46-5
	Detomidine (hydrochloride)	90038-01-0
	Asenapine (maleate)	85650-56-2
Dopamine receptor	Lurasidone (Hydrochloride)	367514-88-3
	Clebopride (malate)	57645-91-7
	Amisulpride	71675-85-9
	rac-Rotigotine (Hydrochloride)	102120-99-0
	Cabergoline	81409-90-7
	ST-836 (hydrochloride)	1415564-68-9
	Azaperone	1649-18-9
	Levosulpiride	23672-07-3
	Rotigotine (Hydrochloride)	125572-93-2
	Chlorprothixene	113-59-7
	Ropinirole (hydrochloride)	91374-20-8

	Paliperidone	144598-75-4
	SKF 38393 (hydrochloride)	62717-42-4
	Iloperidone	133454-47-4
	B-HT 920	36085-73-1
	GBR 12935 (dihydrochloride)	67469-81-2
	Vanoxerine (dihydrochloride)	67469-78-7
Muscarinic acetylcholine (mACh) receptor	Benztropine (mesylate)	132-17-2
	Benzetimide (hydrochloride)	5633-14-7
	Aclidinium (Bromide)	320345-99-1
	Tolterodine (Tartrate)	124937-52-6
	(R)-5-Hydroxymethyl Tolterodine	207679-81-0
	Tiotropium (Bromide)	136310-93-5
	Tarafenacin (D-tartrate)	1159101-48-0
	TBPB	634616-95-8
	Imidafenacin	170105-16-5
	Cevimeline (hydrochloride hemihydrate)	153504-70-2
	Fesoterodine (fumarate)	286930-03-8
	Otilonium (bromide)	26095-59-0
	Diphenmanil (methylsulfate)	62-97-5
	VU0152100	409351-28-6
	Xanomeline (oxalate)	141064-23-5
	Trospium (chloride)	10405-02-4
	Darifenacin (hydrobromide)	133099-07-7
γ-Aminobutyric acid (GABA) receptor	Indiplon	325715-02-4
	Afloqualone	56287-74-2
	Bemegride	64-65-3
	Phenylpiracetam	77472-70-9
	Etifoxine	21715-46-8
	NS11394	951650-22-9
	Lorediplon	917393-39-6
	AWD 131-138	188116-07-6
	Ginkgolide A	15291-75-5
	(R)-Baclofen	69308-37-8
	Dihydroergotoxine (mesylate)	8067-24-1
γ-Secretase	PF-3084014	1290543-63-3
	MK-0752	471905-41-6
	DAPT	208255-80-5

	RO4929097	847925-91-1
	LY-411575	209984-57-6
	BMS-708163	1146699-66-2
	gamma-secretase modulator 3	1431697-84-5
	Semagacestat	425386-60-3
α-Amino-3-hydroxy-5-methyl-4-isoxazolepropionate (AMPA) recptor	Perampanel	380917-97-5
	Talampanel	161832-65-1
	ZK200775	161605-73-8
	CX546	215923-54-9
	Naspm	122306-11-0
	CFM-2	178616-26-7
	PEPA	141286-78-4
	Noopept	157115-85-0
Nicotinic acetylcholine (nACh) receptor	PNU-120596	501925-31-1
	A-867744	1000279-69-5
	Cisatracurium (besylate)	96946-42-8
	PNU-282987	123464-89-1
	Catharanthine	2468-21-5
	Varenicline	249296-44-4
	Vecuronium (bromide)	50700-72-6
Leucine rich-repeat kinase 2 (LRRK2)	PF-06447475	1527473-33-1
	LRRK2-IN-1	1234480-84-2
	GNE-7915	1351761-44-8
	HG-10-102-01	1351758-81-0
	JH-II-127	1700693-08-8
	GSK2578215A	1285515-21-0
	CZC-54252	1191911-27-9
	CZC-25146	1191911-26-8
N-methyl-D-aspartate (NMDA) receptor	Mephenesin	59-47-2
	Eliprodil	119431-25-3
	(+)-MK 801 (Maleate)	77086-22-7
	PEAQX (tetrasodium hydrate)	
	QNZ46	1237744-13-6
	Memantine (hydrochloride)	41100-52-1
	SDZ 220-581	174575-17-8
Opioid receptor	JDTic (dihydrochloride)	785835-79-2
	Trimebutine (maleate)	34140-59-5

	ADL-5859	850173-95-4
	JTC-801	244218-51-7
	Matrine	519-02-8
	Meptazinol (hydrochloride)	59263-76-2
	BAN ORL 24	475150-69-7
Monoamine oxidase	TVP1022	185517-74-2
	Lazabemide	103878-84-8
	Tedizolid (phosphate)	856867-55-5
	Rasagiline (mesylate)	161735-79-1
	Safinamide	133865-89-1
	Tranylcypromine (hemisulfate)	13492-01-8
Neurokinin receptor	Talnetant	174636-32-9
	Fosaprepitant (dimeglumine)	265121-04-8
	SB-222200	174635-69-9
	NKP608	177707-12-9
	Maropitant	147116-67-4
	Aprepitant	170729-80-3
Fatty acid amide hydrolase (FAAH)	PF-3845	1196109-52-0
	FAAH-IN-2	184475-71-6
	URB-597	546141-08-6
	LY2183240	874902-19-9
Selective serotonin reuptake inhibitor (SSRI)	Fluvoxamine (maleate)	61718-82-9
	Dapoxetine (hydrochloride)	129938-20-1
	Sertraline (hydrochloride)	79559-97-0
	Milnacipran (hydrochloride)	101152-94-7
Amyloid-β	Frentizole	26130-02-9
	FPS-ZM1	945714-67-0
	ARN2966	102212-26-0
Acetylcholinesterase (AChE)	Galanthaminone	510-77-0
	(±)-Huperzine A	120786-18-7
Calcitonin receptor-like receptor (CGRP) receptor	MK-3207 (Hydrochloride)	957116-20-0
	MK-0974	781649-09-0
β-Secretase	LY2811376	1194044-20-6
Monoacylglycerol lipase (MAGL)	JZL195	1210004-12-8

Supplementary table 3. Secondary assay results for hit compounds. “↑” indicates that the percentage of Mature Spine Neurons was more than 10% higher than the DMSO treatment group. "↔" denotes that the difference from the DMSO treatment was within ±10%. "↓" denotes that it was more than 10% lower than the DMSO treatment. Each column represents a different compound treatment concentration. Assays are tested at 2-fold (1, 0.5, 0.25, 0.13, 0.06, 0.03 μM) or 3-fold (1, 0.3, 0.1, 0.03, 0.01 μM) lower concentration. Empty columns mean that it is “not evaluated”.

	Compound concentration (μM)								
	0.01	0.03	0.06	0.1	0.13	0.25	0.3	0.5	1
Vortioxetine		↑	↑		↔	↔		↑	↑
8-OH-DPAT	↑	↔		↔			↔		↔
Risperidone	↔	↑		↔			↑		↑
Indacaterol			↑		↑	↑		↑	↔
Tandospirone				↑			↑		↑
Palonosetron				↑			↑		↔
Rotigotine				↔			↔		↑
Diphenmanil				↔			↑		↑
AWD 131-138				↑			↑		↑
A-867744		↔	↑		↔	↑		↑	↔
Cisatracurium		↑		↑			↑		↑
HG-10-102-01		↑		↑			↔		↓
Mephenesin				↔			↔		↑
JTC-801				↑			↑		↑
Maropitant				↑			↑		↔

Supplementary figures

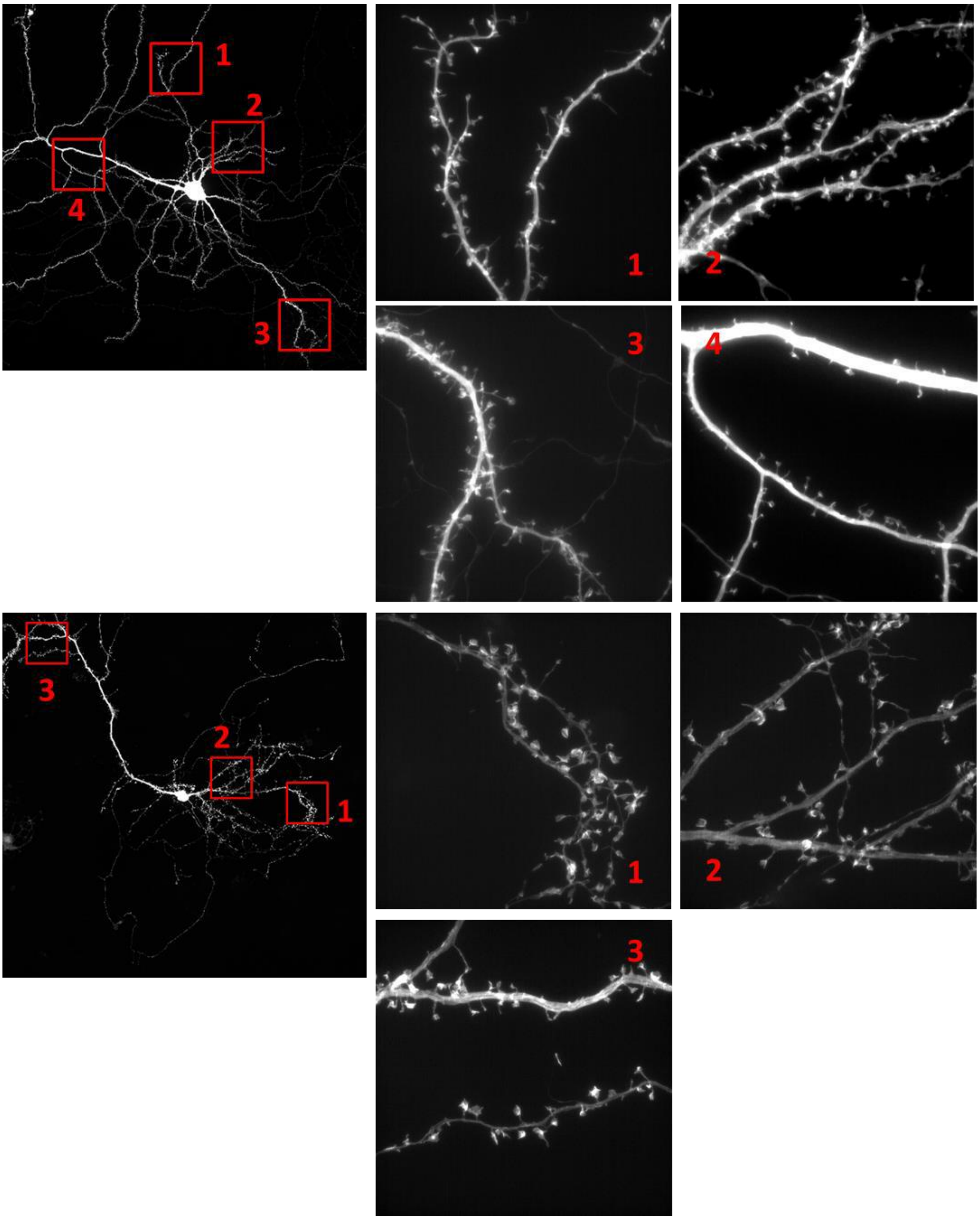

Supplementary figure 1. Visualization of Neuronal Structure Fluorescent images of two cultured neurons from B6 mice that were labeled with the combination of streptavidin-GFP and intracellularly injected biotin using the patch clamp technique. Multiple enlarged images in the red frame are shown in the right panel. The length of one side of the red frame is 40 μm.

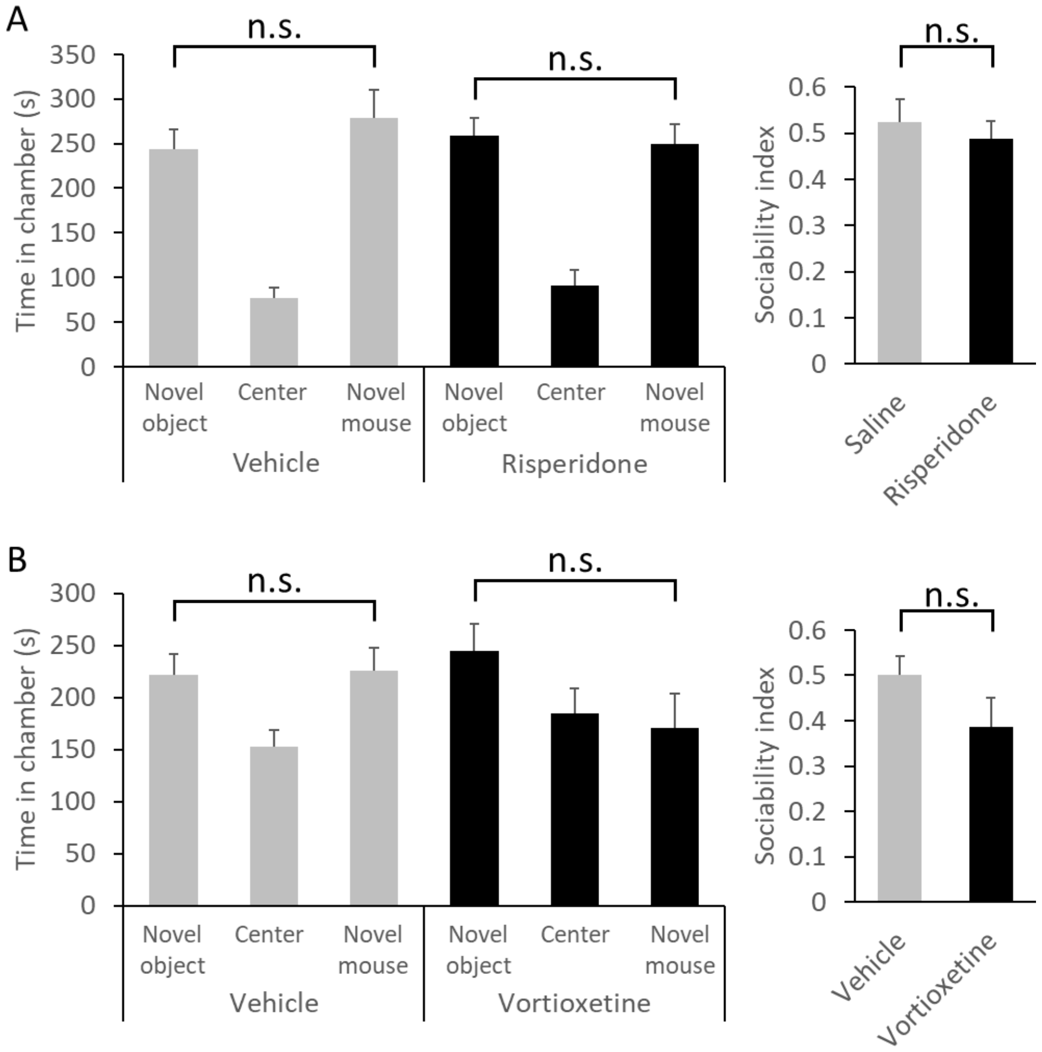

Supplementary figure 2. Efficacy of risperidone and vortioxetine on social behavior in BTBR mice Comparison of social behavior using the 3-chamber social interaction test. Left graph shows time spent in each chamber. Right graph sociability index. (A) Risperidone was administered at a dose of 0.1 mg/kg (i.p) once daily for 7 days. Risperidone was dissolved in saline (0.9% NaCl solution) containing 0.01% v/v acetic acid. (B) Vortioxetine was administered as a single dose at 10 mg/kg (i.p). All bar graph data represent mean ± SEM, *P < 0.05, **P < 0.01, Kruskal-Wallis test with Dunn’s post hoc test and Mann-Whitney U test. N = 13 - 15 /group.
